# EGCG-mediated Protection of the Membrane Disruption and Cytotoxicity Caused by the ‘Active Oligomer’ of α-Synuclein

**DOI:** 10.1038/s41598-017-18349-z

**Published:** 2017-12-20

**Authors:** Jee Eun Yang, Kun Yil Rhoo, Soonkoo Lee, Jong Tak Lee, Jae Hyung Park, Ghibom Bhak, Seung R. Paik

**Affiliations:** 1 School of Chemical and Biological Engineering, Institute of Chemical Processes, College of Engineering, Seoul National University, Seoul, 08826 Republic of Korea; 2 Interdisciplinary program of Bioengineering, College of Engineering, Seoul National University, Seoul, 08826 Republic of Korea

## Abstract

(−)-Epigallocatechin gallate (EGCG), the major component of green tea, has been re-evaluated with α-synuclein (αS), a pathological constituent of Parkinson’s disease, to elaborate its therapeutic value. EGCG has been demonstrated to not only induce the off-pathway ‘compact’ oligomers of αS as suggested previously, but also drastically enhance the amyloid fibril formation of αS. Considering that the EGCG-induced amyloid fibrils could be a product of on-pathway SDS-sensitive ‘transient’ oligomers, the polyphenol effect on the transient ‘active’ oligomers (AOs) was investigated. By facilitating the fibril formation and thus eliminating the toxic AOs, EGCG was shown to suppress the membrane disrupting radiating amyloid fibril formation on the surface of liposomal membranes and thus protect the cells which could be readily affected by AOs. Taken together, EGCG has been suggested to exhibit its protective effect against the αS-mediated cytotoxicity by not only producing the off-pathway ‘compact’ oligomers, but also facilitating the conversion of ‘active’ oligomers into amyloid fibrils.

## Introduction

Parkinson’s disease (PD), the second most prevalent neurodegenerative disease after Alzheimer’s disease, has been characterized by the intra-neuronal Lewy body formation and selective loss of dopaminergic neurons, which results in the hypokinetic manifestations of tremor, rigidity, bradykinesia, and postural instability^1^. α-Synuclein (αS) has been considered to be a pathological component since not only overexpression and point mutations of its gene are demonstrated to be linked to familial early-onset PD, but it is also found to be the major constituent of Lewy bodies by forming the radiating amyloid fibrils^2,3^. Therefore, αS fibrillation process has been focused to unveil its pathological implications although toxic causes leading to the neuronal degeneration still remain to be clarified^4^. In fact, a toxic intermediate hypothesis has suggested that αS oligomers would be a real culprit affecting the cells by disrupting the integrity of membranes via amyloid pore formation whereas the fibrillar end-products simply serve as a detoxification by-product as supported by the fact that the fibril-containing Lewy bodies are often found in the healthy dopaminergic neurons^5,6^.

For the αS oligomers, they are classified into two types. One is ‘transient’ oligomers whose accumulation and subsequent depletion precede the actual amyloid fibril formation^7^. They have been identified in the early lag phase of fibrillation process by employing various techniques such as dynamic light scattering (DLS), Fourier transform infrared spectroscopy (FTIR), fluorescence resonance energy transfer (FRET), small angle X-ray scattering (SAXS), and atomic force microscope (AFM)^7,8^. They are considered to be on-pathway intermediates although it is not clear whether their conversion back to monomers is prerequisite for the fibrillar extension. The other is ‘stable’ oligomers which survive even after the completion of fibrillation^7^. They are thus considered off-pathway oligomers. Several modifications of αS including methionine oxidation and tyrosine nitration gave rise to the ‘stable’ oligomers as a possible non-toxic element^9,10^. In fact, polyphenols such as baicalein and rifampicin have been tested to induce the off-pathway oligomers for the assessment of their therapeutic value^11,12^.

We have also investigated another type of αS oligomers exhibiting rapid self-assembly into the radiating amyloid fibrils (RAFs) on the surface of liposomes, which results in a drastic disruption of the membrane structures^13^. These oligomers characterized as a meta-stable and β-sheet free species, reminiscent of the ‘transient’ oligomers, are named ‘active’ oligomers (AOs) since they instantly turn into amyloid fibrils via a unit-assembly process in which AOs act as a growing unit in the presence of diverse external stimuli such as shear force, temperature change, pH, and organic solvents^14–17^. Since these AOs could be considered as a critical therapeutic target as they are involved in the disintegration of membrane structures, the drug candidates which have been promoted on the basis of their effects on the αS fibrillation may need to be re-evaluated with AOs. Another polyphenol of (−)-epigallocatechin gallate (EGCG), the major flavonoid phytochemical found in green tea, was suggested to effectively suppress the αS fibrillation by stimulating the formation of ‘compact’ oligomers (COs) as the off-pathway product, which led to the reduced cytotoxicity^18^. As a matter of fact, EGCG has been known as the most effective radical scavenger exhibiting anti-oxidative and anti-inflammatory properties^19^. It was also suggested that EGCG could prevent several pathological disorders including cardiovascular disease, cancer, and neurodegenerative diseases with its daily intake^19^. Furthermore, a long-term treatment with EGCG was shown to not only increase the lifespan, but also improve the movement deficit observed with a *Drosophila melanogaster* model of PD^20^. In this report, therefore, EGCG has been investigated with the AOs of αS in terms of its effects on the unit-assembly process of AOs leading to the fibril formation and the membrane disruption contributing to cytotoxicity.

## Results and Discussion

### Molecular interaction between αS and EGCG

**Figure 1:**
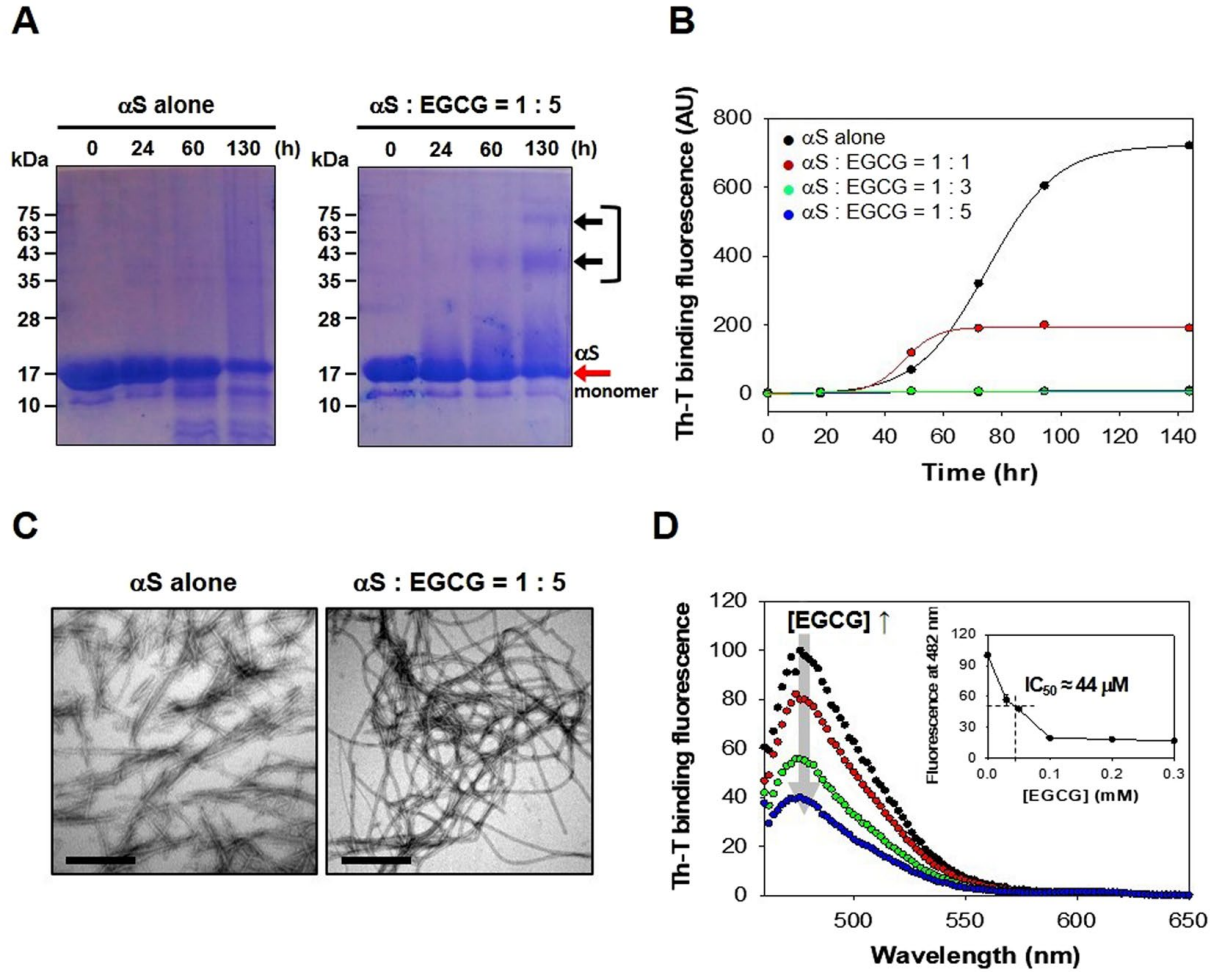
Analyses of molecular interaction between αS and EGCG. (**A**) Time-resolved SDS-PAGE analyses of the αS fibrillation carried out in the absence or presence of EGCG. The SDS-resistant aggregates of αS were revealed as the protein bands indicated with black arrows. Full-length gels are presented in Supplementary Information Figure 6. (**B**) Fibrillations of αS (70 μM) in the presence of various concentrations of EGCG at molar ratios indicated were monitored with Th-T binding fluorescence assay. (**C**) TEM images of αS amyloid fibrils prepared without and with EGCG. Scale bars represent 0.5 μm. αS and EGCG were co-incubated for 130 hr at 37 °C with agitation. (**D**) Interference of EGCG with the Th-T binding fluorescence of amyloid fibrils. The Th-T treated amyloid fibrils were co-incubated with various concentrations of EGCG (0 μM for black dots, 70 μM for red dots, 210 μM for green dots, 350 μM for blue dots) for 5 min. Inset shows the Th-T binding fluorescence intensity monitored at 482 nm in the presence of various EGCG concentrations.

Molecular effect of EGCG on the self-assembly of αS was evaluated with 15% SDS-PAGE by analyzing the final products of αS (70 μM) incubated in the absence or presence of 350 μM EGCG with agitation for up to 130 hr at 37 °C (Fig. 1A). After Coomassie Brilliant Blue (CBB) staining, the SDS-resistant species of αS oligomers were revealed as the discrete bands at higher molecular weights of 43 and 75 kDa than monomeric αS at 17 kDa, which were found only in the co-incubated mixture between αS and EGCG (Fig. 1A, right). αS alone, however, did not give rise to the oligomeric species even after 130 hr of an extensive incubation, but produced a steak of protein smear spanning the entire lane above the αS monomer band (Fig. 1A, left). In fact, the SDS-resistant species were previously suggested to be derived from the EGCG-induced COs of αS^18^. According to this study, EGCG was demonstrated to induce the αS COs while preventing the fibrillation of αS as determined mainly with the reduced Th-T binding fluorescence. As a matter of fact, our experiment also confirmed the decrease in the Th-T binding fluorescence of the co-incubated mixtures of αS and EGCG at various concentrations during 140 hr of incubation at 37 °C (Fig. 1B). The final Th-T binding fluorescence decreased drastically as the EGCG level increased. In the presence of 210 μM EGCG at three-fold molar excess to αS, the fluorescence disappeared completely.

Intriguingly, however, the co-incubated mixture of αS with EGCG at 1:5 ratio yielding completely diminished Th-T binding fluorescence at the completion of fibrillation revealed a decent network of amyloid fibrils examined under TEM (Fig. 1C, right), which appeared slightly curly compared to the straight fibrils obtained with αS incubated alone in the absence of EGCG (Fig. 1C, left). This result clearly indicated that EGCG did not inhibit the αS fibrillation but actually facilitate it, which also contradicts another observation that EGCG acts as a remodeling agent favoring the production of small amorphous protein aggregates from mature αS fibrils^18,21^. In fact, EGCG was found to simply interfere with the Th-T binding fluorescence of amyloid fibrils by directly binding to the β-sheet structure^22^. To evaluate the interference, the pre-made αS fibrils (7 μM) were incubated with Th-T (2.5 μM) in the presence of various concentrations of EGCG for 5 min, and their emission spectra were monitored with an excitation at 450 nm (Fig. 1D). The result indicated that the Th-T binding fluorescence peak observed at 482 nm decreased as the concentration of EGCG increased with a half maximal inhibitory concentration (IC_50_) of 44 μM (Fig. 1D, *inset*). Additionally, it was also confirmed that this obvious decrease in Th-T binding fluorescence was not caused by either EGCG-induced disintegration or structural alteration of amyloid fibrils as assessed with DLS, TEM, CD, and FTIR analyses (Supplementary Information Figure S1). Therefore, the Th-T binding fluorescence is found to be inappropriate to evaluate the ‘inhibitory’ effect of EGCG toward the αS fibrillation^18,22^.

**Figure 2:**
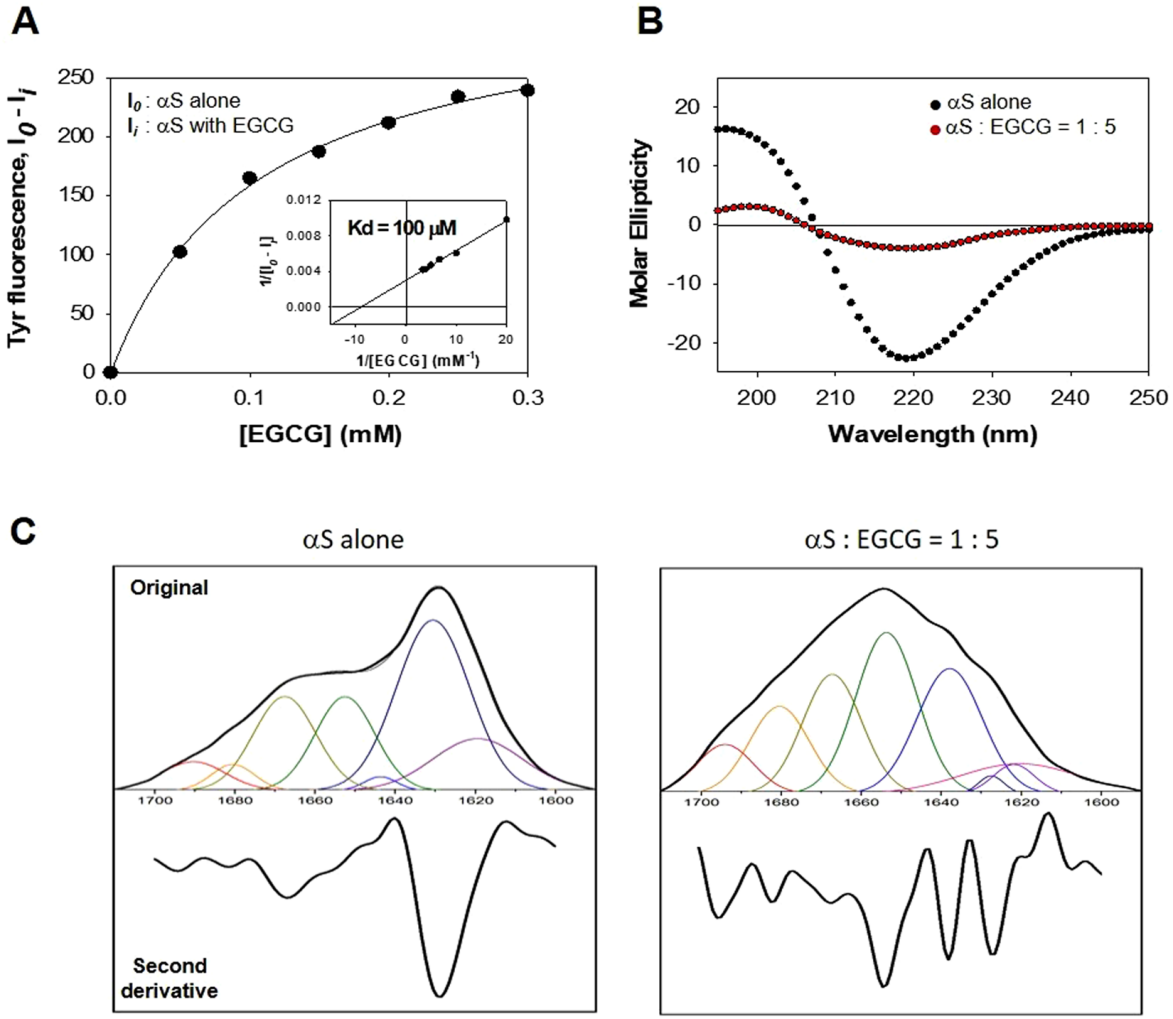
Spectroscopic evaluation of molecular interaction between αS and EGCG with intrinsic fluorescence, CD spectroscopy, and FTIR spectroscopy. (**A**) Dissociation constant between αS and EGCG assessed with the intrinsic fluorescence of tyrosine residues of αS. Differences in the tyrosine intrinsic fluorescence emitted at 308 nm in the presence (I*i*) and absence (I*o*) of EGCG were plotted as a function of EGCG concentration. Dissociation constant (K_d_) of 100 μM was calculated from a double-reciprocal plot of the saturation curve (Inset). (**B**) Circular dichroism (CD) spectra of the amyloid fibrils of αS prepared without (black dots) and with (red dots) EGCG following co-incubation for 130 hr at 37 °C with agitation. (**C**) FTIR spectra and their second derivative spectra of the amyloid fibrils of αS prepared without (left) and with (right) EGCG after 96hr incubation at 37 °C with agitation. The FTIR absorption spectra have been deconvolved according to the peaks found in the second derivative spectra.

Nevertheless, since the SDS-resistant oligomers and the curly fibrils of αS were evidently induced by EGCG, the apparent molecular interaction between αS and EGCG was evaluated by monitoring intrinsic fluorescence of tyrosine residues of αS (Supplementary Information Figure S2). Their specific molecular interaction was verified with a hyperbolic curve obtained by plotting the difference in the intrinsic fluorescence observed at 308 nm in the absence and presence of EGCG as a function of EGCG concentration (Fig. 2A). A double reciprocal plot of the hyperbolic curve provided a dissociation constant (K_d_) of 100 μM between EGCG and monomeric αS (Fig. 2A, inset). Unfortunately, however, the actual EGCG binding site of αS has not been identified. A few studies employing NMR suggested that EGCG would bind to the highly flexible C-terminal region of αS, which led to compaction of αS monomers and immobilization of the flexible C-terminus in the αS oligomers^18,23–25^. As the EGCG level increased, however, the compound was demonstrated to bind αS nonspecifically to the backbone groups rather than a specific area^18^, which was also confirmed by native top-down electron transfer dissociation (ETD) experiments employing nano electrospray-ionization mass spectrometry^24^.

Nonetheless, the molecular interaction resulted in the amyloid fibrils of αS distinctive from those obtained in the absence of EGCG as assessed with CD spectra in which a typical β-sheet conformation of amyloid fibrils yielding the single minimum ellipticity at 220 nm became far less obvious (Fig. 2B). For the EGCG-induced amyloid fibrils (EGCG-AFs), the minimum at 220 nm was raised considerably while the maximum at 200 nm decreased, which might indicate a possible random structure formation in addition to altered β-sheet structure. To elaborate the nature of EGCG-AFs, therefore, FTIR was employed to examine the fibrils. In comparison with the control of αS amyloid fibrils prepared in the absence of EGCG, a distinctive spectrum deconvolved based on the second derivative spectrum was obtained (Fig. 2C), in which two peaks located at 1680 and 1650 cm^−1^ became apparently increased while the main peak at 1630 cm^−1^ decreased. The two components at 1630 and 1680 cm^−1^ have been used to designate the β-pleated sheet structure^26^. The increase in the high-frequency component with the concomitant decrease in the low-frequency component, therefore, could indicate the presence of anti-parallel β-sheet structure in the EGCG-AFs. In addition, a decent increase in the random structure was also realized for the final aggregates of αS induced by EGCG as determined by the increased peak at 1650 cm^−1^ although it was not clear whether the random component was an intrinsic part of the fibrils or due to the COs shown to co-exist with the EGCG-AFs (See below). Taken together, EGCG can be considered as a relatively selective αS-interactive small molecule inducing the SDS-resistant oligomers of αS and causing its fibrillar polymorphism.

### Two distinctive types of αS oligomers produced by EGCG

**Figure 3:**
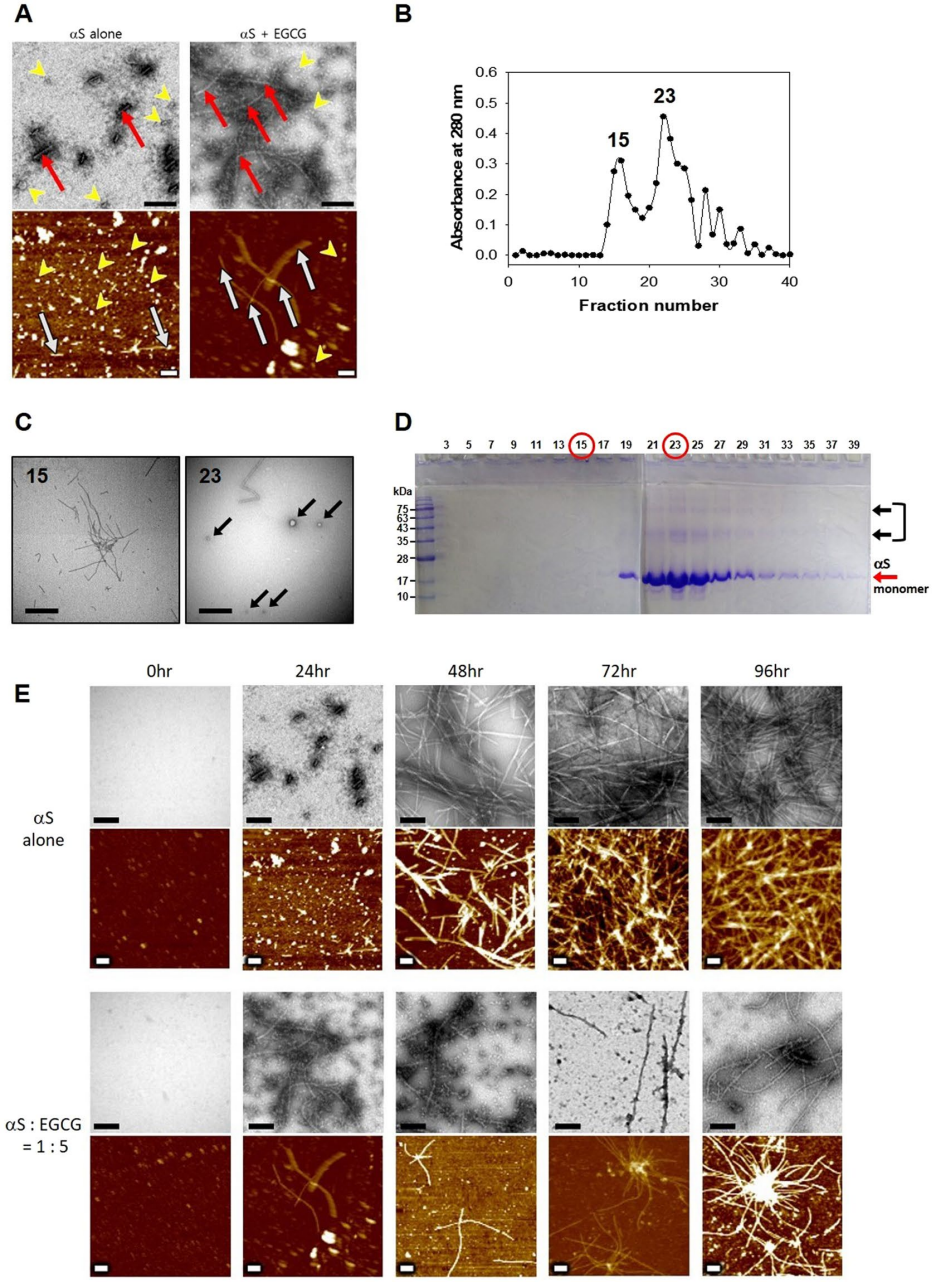
Co-existence of the two distinctive types of αS aggregates produced by EGCG. (**A**) The oligomers (yellow arrow heads) and amyloid fibrils (red or white arrows) of αS (70 μM) obtained after incubation with and without EGCG (350 μM) at 37 °C for 24 hr with agitation were revealed with TEM (upper) and AFM (lower). Scale bars represent 200 nm. (**B**) Separation of the oligomers and amyloid fibrils with a size exclusion chromatography (Sephacryl S-200). (**C**) TEM images of the aggregates present in the fractions numbered 15 and 23 from the chromatography. The granular forms of oligomeric species are indicated with black arrows. Scale bars indicate 0.5 μm. (**D**) SDS-PAGE analysis of the fractions from the size exclusion chromatography. The gels were stained with CBB. The SDS-resistant aggregates of αS were revealed as the high molecular weight protein bands indicated by black arrows. (**E**) The αS aggregation processes occurring in the absence (upper) and presence (lower) of EGCG at 1:5 molar ratio were followed with the images of TEM and AFM. Scale bars represent 200 nm.

Based on the observations described above, it is pertinent to consider that EGCG induces two types of αS oligomers. One is the off-pathway COs which are SDS-resistant and no longer participating in the amyloid fibril formation. The other is ‘transient’ oligomers suggested to be SDS-sensitive and prone to grow into amyloid fibrils^7^. In fact, upon EGCG treatment, αS ended up producing not only COs but also amyloid fibrils as revealed with TEM and AFM (Fig. 3A). Those COs and amyloid fibrils were separated via Sephacryl S-200 size-exclusion chromatography from the 24-hr incubated mixture of αS and EGCG (Fig. 3B). Among all the collected fractions, two major peaks culminated at fraction number 15 and 23 were shown to contain the amyloid fibrils and the COs, respectively, as revealed with TEM (Fig. 3C). Furthermore, SDS-PAGE analysis also confirmed the presence of the two separate species of αS pre-treated with EGCG (Fig. 3D). Since no protein band was observed in the lane of fraction number 15, the EGCG-induced amyloid fibrils (EGCG-AFs) were obviously resistant to SDS. On the other hand, several discrete protein bands comprising monomeric αS and a few higher molecular weight species were visualized from the fraction 23. Whereas the 17 kDa monomeric αS could be a product of SDS-sensitive ‘transient’ oligomers, the presence of higher molecular weight species such as 43 kDa and 75 kDa was another indication of the formation of SDS-resistant COs of αS upon the EGCG treatment. To clarify the co-existence of COs and EGCG-AFs, the αS fibrillation kinetics was followed with TEM and AFM in the absence and presence of EGCG at 1:5 molar ratio (αS: EGCG) (Fig. 3E and Supplementary Information Figure S3). Based on the images of TEM and AFM obtained after a prolonged incubation for 96 hours, EGCG was shown to transform αS into two separate pools of the COs and EGCG-AFs whereas αS alone produced amyloid fibrils as an exclusive product (Fig. 3E). Their co-existence became evident from 48 hours and continued to the end of incubation (Fig. 3E). In fact, the EGCG-AFs actually emerged earlier during the initial 24 hours of incubation while αS itself produced only the oligomers in the absence of EGCG (Supplementary Information Figure S3). These data clearly indicate that αS has been transformed into the two separate pools of the COs and amyloid fibrils in the presence of EGCG.

### EGCG effects on the AOs of αS

**Figure 4:**
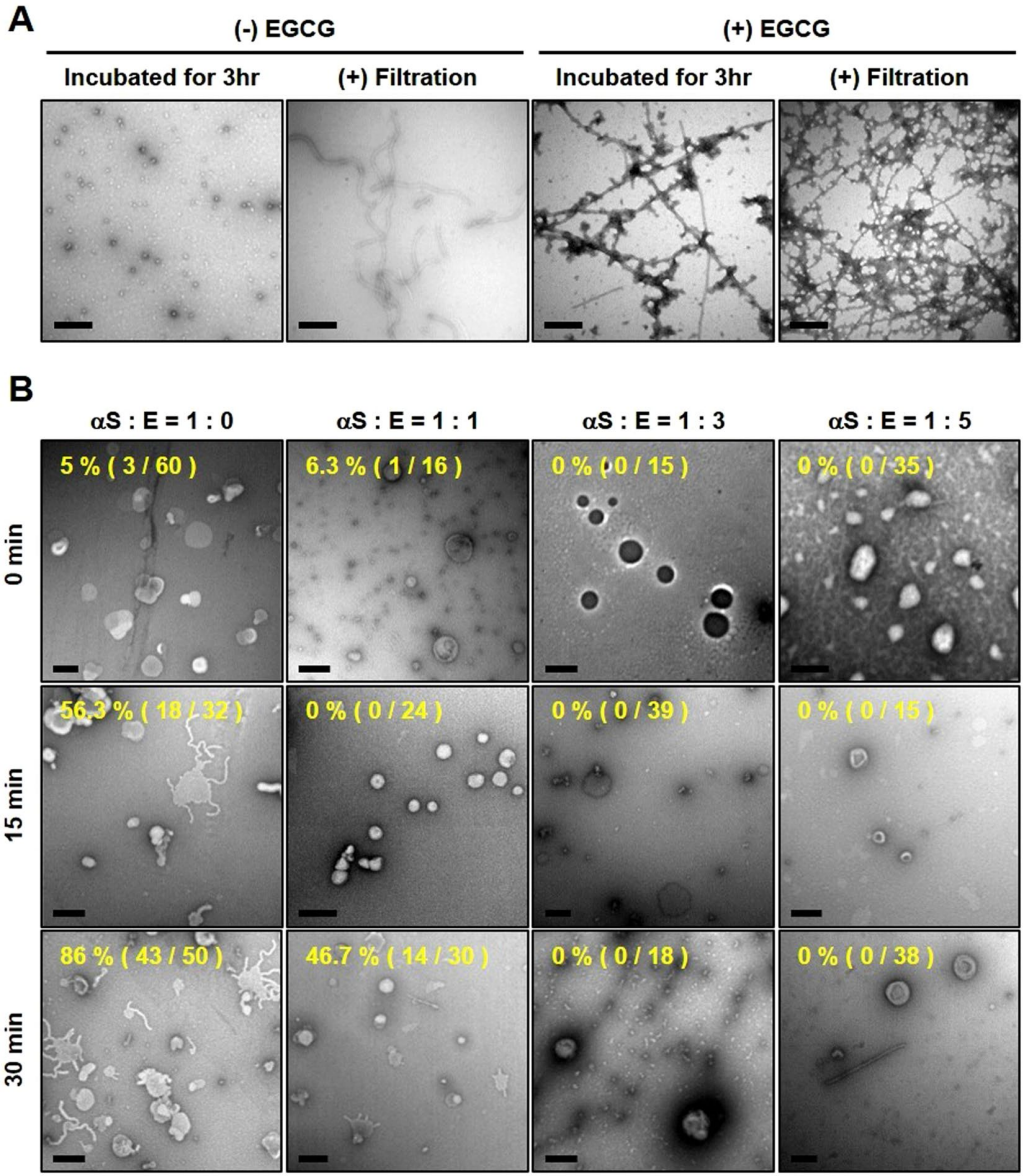
EGCG effects on the ‘active’ oligomers (AOs) of αS. (**A**) Production of CAFs from AOs (70 μM) via the repetitive centrifugal membrane filtration in the presence or absence of EGCG (350 μM) was assessed with TEM. Scale bars represent 0.5 μm. (**B**) Membrane disruption by AOs. Following the pre-incubation of AOs (70 μM) with EGCG at various molar ratios for 3 hr at 37 °C, the DOPC-liposomes were treated with the AOs for 0 min, 15 min, and 30 min. The membrane disruption and RAF formation were examined with TEM. Percentages of the disrupted liposomes are indicated. Scale bars represent 200 nm.

Besides the off-pathway COs, therefore, the EGCG-AFs could be resulted from the SDS-sensitive ‘transient’ oligomers of αS. Since the ‘transient’ oligomers have shared their core characteristics with the AOs of αS, which include the meta-stable property as evidenced by SDS-sensitivity and their conversion into polymorphic amyloid fibrils under the influence of chemical and mechanical stimuli^15,17^, EGCG effects on the AOs were investigated in terms of their effects on the unit-assembly into amyloid fibrils and the radiating amyloid fibril (RAF) formation on the surface of liposomal membranes. The AOs which remained unaffected during 3 hr incubation at 37 °C in the absence of EGCG turned into the curly amyloid fibrils (CAFs) following the repetitive membrane filtration as previously demonstrated (Fig. 4A, left)^15,27^. In the presence of EGCG, almost all the AOs became assembled into the fibrils with some fibril-associated aggregates during the initial 3 hr incubation. In addition, the membrane filtration failed to produce CAFs, indicating that ECGC has facilitated the fibril formation of AOs with distinctive morphology (Fig. 4A, right). The RAF formation of AOs on the surface of DOPC liposomes was also examined with TEM following the pre-incubation of AOs with various concentrations of EGCG (Fig. 4B). In the absence of EGCG, RAFs were instantly developed on the liposomes even after 15 min of incubation with AOs, which resulted in a drastic structural disruption of the membranes as reported previously^13^. In fact, 86% of the liposomes were disrupted with considerable RAF formation after 30 min of the incubation. In the presence of AOs pre-treated with 70 μM EGCG at 1:1 molar ratio with αS, the liposomal disruption decreased to 47% with noticeably reduced RAF formation during the 30 min incubation. As the EGCG level increased during the pre-incubation with AOs, the RAF formation became scarce, which left the liposomes unaffected with the presence of separate pools of the granular aggregates and fibrils. Therefore, it is pertinent to suggest that EGCG certainly deprives the AOs of the membrane surface-dependent self-assembly property leading to the RAF formation by facilitating their conversion to amyloid fibrils as a non-toxic element. This observation is discriminated from another previous suggestion that EGCG prevents the membrane disruption by binding to the αS oligomers and thus interfering with the oligomer-membrane interaction^23^. Although both studies have reached to a similar conclusion that EGCG would suppress the cytotoxicity caused by αS oligomers exhibiting vesicle destabilization rather than membrane pore formation, the actual means of membrane destabilization caused by the two types of oligomers are suggested to be distinctive. The AOs investigated in this study destabilize the membranes by producing the RAFs on the surface of DOPC liposomes while the other type of oligomers have disrupted the liposomes of DOPG (1,2-dioleoyl-sn-3-phosphatidylglycerol) through vesicle permeabilization. In the presence of EGCG, however, the AOs in this study have lost their cytotoxic activity via the facilitated transformation into the amyloid fibrils and COs whereas the other oligomers became less effective on the membrane binding without altering the size distribution and the secondary structure of the oligomers^23^. These distinctive responses of the αS oligomers toward EGCG could be due to disparate initial states of the oligomers prepared in different incubation conditions. The presence of NaCl, for example, might hinder a pristine molecular interaction between αS oligomers and EGCG.

### EGCG suppresses the cytotoxicity caused by AOs of αS

**Figure 5:**
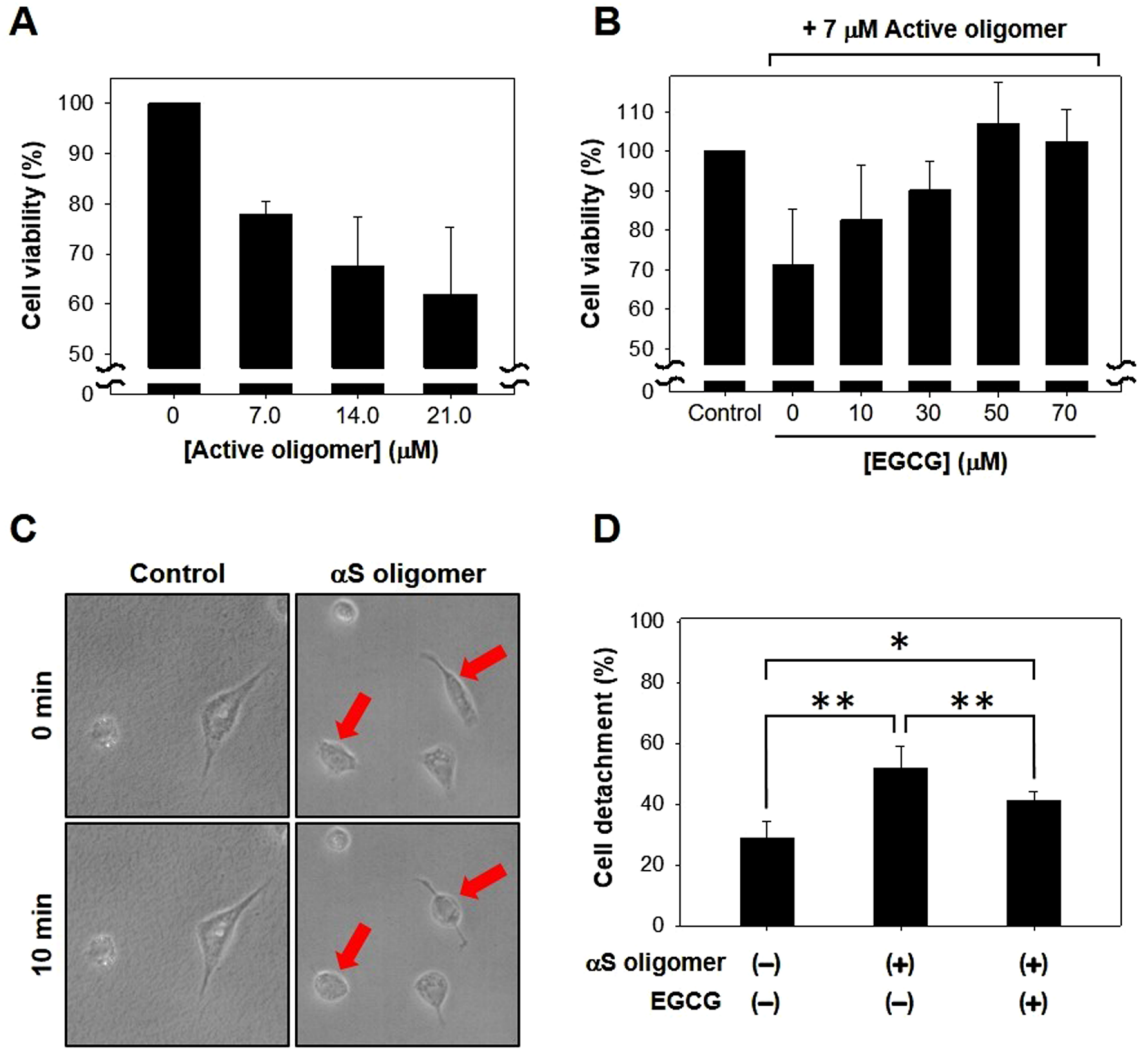
EGCG effect on the cytotoxicity caused by AOs. (**A**) Cytotoxicity caused by AOs. SH-SY5Y cells were treated with AOs at various concentrations for 4 hr at 37 °C. The cell viability was assessed with colorimetric MTS assay. (**B**) Cell viability of SH-SY5Y cells after 4 hr incubation with the AOs (7 μM) pre-treated with EGCG at various concentrations. (**C**) The cells attached on a glass slide were treated with AOs at a high concentration of 70 μM for 10 min at room temperature. The arrows indicate the morphologically altered cells on the surface. (**D**) Cell detachment from a glass surface. After treating SH-SY5Y cells with the AOs (70 μM) pre-treated with and without EGCG (350 μM) for 3 hr, the cells detached from the surface before and after the 10 min incubation were counted and compared. *and **indicate t-test with p ≤ 0.05 and p ≤ 0.01, respectively, between the groups compared.

Since the RAF-mediated membrane disruption of AOs has been suggested to be responsible for cellular degeneration^13^, EGCG effect on the cytotoxicity induced by the AOs was examined *in vitro*. Human dopaminergic neuroblastoma cells (SH-SY5Y) were incubated with the AOs pre-treated with or without EGCG. MTS assay indicated that the cell viability gradually decreased as the AOs’ level increased (Fig. 5A). However, the AO-mediated cytotoxicity was deterred by a prior EGCG-treatment of the AOs. As the EGCG concentrations increased during the pre-treatment, the cell viability assessed with MTS assay also increased from 72% to 102% in the absence and presence of 70 μM EGCG, respectively (Fig. 5B). In addition, the AO effect was also evaluated with the cells in terms of their shape change from an elongated surface-stabilized state to a spherical form detached from a glass surface after 10 min of the AO treatment at 70 μM **(**Fig. 5C). In fact, the cell detachment was suppressed by the EGCG pre-treatment of AOs (Supplementary Information Figure S4). While the AOs alone caused the cells readily detached by more than 50% after a short period of incubation for 10 min from a control level of 30% without the AOs, the cell detachment was prevented to 40% in the presence of the EGCG-treated AOs (Fig. 5D). Taken together, therefore, EGCG has been suggested to exhibit its protective effect against the αS-mediated cytotoxicity by not only producing the off-pathway COs^18^ but also facilitating the conversion of AOs into amyloid fibrils and thus accelerating the removal of AOs which could exert the membrane disruption and subsequent cellular degeneration if untouched.

Based on the data provided in this report, it is pertinent to consider that EGCG as the key component of green tea would be a possible prophylactic or even therapeutic agent toward PD by either suppressing a population of toxic AOs or promoting the non-toxic CO formation of αS. To be more definitive, however, these oligomers need to be elaborated in terms of their toxic activity and pathological involvement since a diverse set of αS oligomers have been investigated with different molecular and cellular activities and their mutual relationships are still required to be clarified.

## Materials and Methods

### Materials

Carbon-coated copper grid was purchased from Ted Pella Inc (Redding, CA). DNase, DEAE-Sephacel, (−)-Epigallocatechin gallate (EGCG), isopropylthiogalactoside (IPTG), lysozyme, 2-(N-morpholino)ethanesulfonic acid (Mes), 3-(4,5-dimethylthiazol-2-yl)-5-(3-carboxymeth-oxyphenyl)-2-(4-sulfophenyl)-2H-tetrazolium (MTS), Sephacryl S-200, S-Sepharose, and thioflavin-T (Th-T) were purchased from Sigma-Aldrich (St. Louis, MO). 1,2-Dioleoyl-sn-glycero-3-phosphocholine (DOPC) was obtained from Avanti Polar Lipids Inc (Alabaster, AL). LB medium was purchased from Becton Dickinson & Company (San Diego, CA), and uranyl acetate was obtained from Electron Microscopy Sciences (Hatfield, PA). Microcon YM30 was provided by Merck Millipore (Billerica, MA). Dimethyl cyclodextrin was supplied from the Microbial Carbohydrate Resource Bank at Konkuk University.

### Preparation of αS ‘active’ oligomers (AOs)

Human recombinant α-synuclein (αS) was expressed in *E. coli* BL21 (DE3) and purified according to the procedure reported previously^28^. Briefly, the transformed cells were cultured in LB medium and the gene expression was induced by adding 0.5 mM IPTG. αS, extracted from the cells through a freezing-thawing process in the lysis buffer containing lysozyme and DNase, was purified via three successive chromatography steps of anion exchange (DEAE-Sephacel), size-exclusion (S-200), and cation-exchange (S-Sepharose) chromatography. After a dialysis against total 6 L of 20 mM Mes at pH 6.5 with two changes, the purified αS was obtained and stored in aliquots at −80 °C. The AOs were prepared by incubating monomeric αS (70 μM) for 6 hr at 37 °C in 20 mM Mes (pH 6.5) under a shaking condition at 200 rpm. According to the previous reports^14,22^, the AOs were confirmed with two criteria of their granular homogeneity assessed with transmission electron microscope (TEM) and a lack of β-sheet content monitored with circular dichroism (CD) spectroscopy (Supplementary Information Figure S5). In addition, the CAF formation with centrifugal membrane filtration and the RAF formation on the surface of DOPC liposomes were also examined to confirm the authenticity of AOs (Supplementary Information Figure S5).

### Amyloid fibril formation with and without EGCG

In the absence and presence of EGCG at various concentrations, the progress of αS (70 μM) fibrillation was monitored with amyloid-specific Th-T binding fluorescence. In detail, aliquots (20μl) of the αS incubated in 20 mM Mes (pH 6.5) at 37 °C under a shaking condition at 200 rpm were taken at various time points during the fibrillation and incubated with 2.5μM Th-T in 50 mM glycine (pH 8.5) for 5 min at room temperature. The Th-T binding fluorescence at 485 nm with an excitation at 450 nm was measured with a chemiluminescence spectrophotometer (LS-55B, Perkin-Elmer). In addition, centrifugal membrane filtration was also employed to prepare the CAFs from the AOs by using Microcon YM30^15^. Following repetitive membrane filtrations at 14,000 × g for 5 times with 10 min each, the resulting fibrils were examined with TEM.

### Tyrosine intrinsic fluorescence assay

After incubating αS (70 μM) with and without various concentrations of EGCG for 30 min at 37 °C, the intrinsic tyrosine fluorescence of αS was monitored between 300 nm and 400 nm with excitation at 274 nm with a spectrophotometer (LS-55B, Perkin-Elmer). Saturation curve was produced by plotting the difference in the intrinsic fluorescence obtained in the absence (I_0_) and presence (I_i_) of EGCG as a function of EGCG concentration. Dissociation constant K_d_ was obtained from the x-intercept of a double-reciprocal plot between 1/[EGCG] and 1/(I_0_ − I_i_) for x- and y-axis, respectively.

### Transmission electron microscopy (TEM)

An aliquot (20 μL) of the samples containing protein aggregates was adsorbed onto a carbon-coated copper grid (200-mesh, Electron Microscopy Sciences) and air-dried for 3 min. Following a negative staining with 2% uranyl acetate for 5 min, the grids containing the αS-related structures were examined with TEM (JEM1010, JEOL).

### Circular dichroism (CD) spectroscopy

αS (70 μM) fibrils prepared without and with EGCG (350 μM) were precipitated via centrifugation, and resuspended in 0.5 mL of distilled water. Protein secondary structure of the samples were analyzed with CD spectroscopy (Chirascan-plus, Applied Photophysics) between 195 and 260 nm with a scan speed of 10 nm/min.

### Dynamic light scattering (DLS)

Size distribution of αS amyloid fibrils treated with and without EGCG was monitored by using DLS (Zetasizer Nano ZS, Malvern) at 25 °C. The amyloid fibrils of αS were incubated with 0, 0.1, and 0.3 mM EGCG at 37 °C in an orbital shaker for 1 hr. Following the incubation, the mixtures of amyloid fibrils and EGCG were collected via a centrifugation at 16,100 $\times$ g for 30 min and the collected pellets were resuspended with 1 mL of distilled water for DLS analysis.

### Fourier transform infrared (FTIR) spectroscopy

The precipitates of amyloid fibrils prepared in the absence or presence of EGCG after solvent evaporation with lyophilization were placed on ZnSe ATP crystal and ATR spectra were monitored with Nicolet 6700 FTIR spectrometer equipped with DTGS detector (Thermo Fisher Scientific Inc.). Data fitting and curve deconvolution of the ATR-FTIR spectra were performed based on the frequencies of the peaks found in the second-derivative spectra by using OriginPro 2015 software (OriginLab, Northampton, MA).

### Atomic force microscopy (AFM)

Ten μL of αS (70 μM) incubated with and without EGCG (350 μM) at 37 °C under a shaking condition (200 rpm) was loaded onto a slide glass plate (Marienfeld, Germany) coated with 0.1% poly-L-lysine (w/v) and left to be fixed for 5 min. The glass plate was rinsed gently with distilled water several times and dried in a vacuum chamber. The assembled structures of αS were examined with Atomic Force Microscopy (NX-10, Park system, Korea) operating with non-contact cantilever (PPP-NCHR 10 M, Park System, Korea).

### Liposome preparation

Homogenous DOPC (1 mg/mL) solution in chloroform was prepared in a test tube and then lipid film was formed on the surface of the test tube following evaporation of chloroform with N_2_ gas. After complete removal of chloroform by being stored in a desiccator overnight, the lipid film was heated in a water bath at 80 °C for 1 min and incubated with 1 mL of 20 mM Mes (pH 6.5) at 60 °C for 1 hr under a shaking condition at 300 rpm. The liposomes were finally prepared by extruding the hydrated lipid solution at 60 °C through a polycarbonate membrane (pore size of 200 nm) equipped in an extruder set (Avanti Polar Lipids Inc.).

### Cell viability assessment with MTS assay

Human dopaminergic neuroblastoma cells (SH-SY5Y) were seeded in a 96-well microtiter plate at ~1.0 × 10^6^ cells/well. After the cell confluency reached to about 90%, the mixtures of AOs (7μM) and EGCG at various concentrations incubated together for 3hr at 37 °C under a shaking condition (200 rpm) were added and further incubated for 24 hr in 5% CO_2_ at 37 °C. In order to measure the cell proliferation, 20 μL of MTS solution was added to the wells and incubated for 4 hr. Following termination of the reaction by adding 25 μL of 10% SDS, the absorbance of each well was measured at 490 nm with a microplate spectrophotometer (Multiskan^TM^ GO, Thermo Fisher Scientific Inc.).

### Statistical Analysis

Data analyses were performed using t-test for two independent means with a two-tailed hypothesis. For each statistical analysis, *n* = 4 and results are presented as mean ± standard deviation (SD). α-Levels used for comparison tests are 0.1 for the test between control group and AO-treated group (‘AO group’) and the test between ‘AO group’ and the group of AO pre-incubated with EGCG (‘EGCG group’) and 0.5 for the test between control group and ‘EGCG group’. Actual p values for the tests are as follows: 0.008659 for the test between control group and ‘AO group’, 0.003144 for the test between ‘AO group’ and ‘EGCG group’, and 0.025036 for the test between control group and ‘EGCG group’.

### Data availability

All data generated or analyzed during this study are included in this published article and its Supplementary Information file.

## Electronic supplementary material

Supplementary information

## References

[1] Goedert M (2001). a-synuclein and neurodegenerative diseases. Nat. Rev. Neurosci..

[2] Polymeropoulos MH (1997). Mutation in the a-synuclein gene identified in families with Parkinson’s disease. Science.

[3] Spillantini MG, Crowther RA, Jakes R, Hasegawa M, Goedert M (1998). a-synuclein in filamentous inclusions of Lewy bodies from Parkinson’s disease and dementia with lewy bodies. Proc. Natl. Acad. Sci. USA.

[4] Sacchettini JC, Kelly JW (2002). Therapeutic strategies for human amyloid diseases. Nat. Rev. Drug Discov..

[5] Lashuel HA (2002). a-synuclein, especially the Parkinson’s disease-associated mutants, forms pore-like annular and tubular protofibrils. J. Mol. Biol..

[6] Volles MJ (2001). Vesicle permeabilization by protofibrillar a-synuclein: implications for the pathogenesis and treatment of Parkinson’s disease. Biochemistry.

[7] Fink AL (2006). The aggregation and fibrillation of a-synuclein. Acc. Chem. Res..

[8] Kaylor J (2005). Characterization of oligomeric intermediates in a-synuclein fibrillation: FRET studies of Y125W/Y133F/Y136F a-synuclein. J. Mol. Biol..

[9] Glaser CB, Yamin G, Uversky VN, Fink AL (2005). Methionine oxidation, a-synuclein and Parkinson’s disease. Biochim. Biophys. Acta..

[10] Yamin G, Uversky VN, Fink AL (2003). Nitration inhibits fibrillation of human a-synuclein *in vitro* by formation of soluble oligomers. FEBS Lett..

[11] Li J, Zhu M, Rajamani S, Uversky VN, Fink AL (2004). Rifampicin inhibits a-synuclein fibrillation and disaggregates fibrils. Chem. Biol..

[12] Zhu M (2004). The flavonoid baicalein inhibits fibrillation of a-synuclein and disaggregates existing fibrils. J. Biol. Chem..

[13] Lee JH (2012). Radiating amyloid fibril formation on the surface of lipid membranes through unit-assembly of oligomeric species of a-synuclein. PLoS One.

[14] Bhak G (2014). Molecular inscription of environmental information into protein suprastructures: temperature effects on unit assembly of a-synuclein oligomers into polymorphic amyloid fibrils. Biochem. J..

[15] Bhak G, Lee JH, Hahn JS, Paik SR (2009). Granular assembly of a-synuclein leading to the accelerated amyloid fibril formation with shear stress. PLoS One.

[16] Lee D (2011). Photoconductivity of pea-pod-type chains of gold nanoparticles encapsulated within dielectric amyloid protein nanofibrils of a-synuclein. Angew. Chem. Int. Ed..

[17] Lee JH, Bhak G, Lee SG, Paik SR (2008). Instantaneous amyloid fibril formation of a-synuclein from the oligomeric granular structures in the presence of hexane. Biophys. J..

[18] Ehrnhoefer DE (2008). EGCG redirects amyloidogenic polypeptides into unstructured, off-pathway oligomers. Nat. Struct. Mol. Biol..

[19] Singh BN, Shankar S, Srivastava RK (2011). Green tea catechin, epigallocatechin-3-gallate (EGCG): mechanisms, perspectives and clinical applications. Biochem. Pharmacol..

[20] Ortega-Arellano HF, Jimenez-Del-Rio M, Velez-Pardo C (2011). Life span and locomotor activity modification by glucose and polyphenols in Drosophila melanogaster chronically exposed to oxidative stress-stimuli: implications in Parkinson’s disease. Neurochem. Res..

[21] Bieschke J (2010). EGCG remodels mature α-synuclein and amyloid-β fibrils and reduces cellular toxicity. Proc. Natl. Acad. Sci. USA.

[22] Hudson SA, Ecroyd H, Kee TW, Carver JA (2009). The thioflavin T fluorescence assay for amyloid fibril detection can be biased by the presence of exogenous compounds. FEBS J..

[23] Lorenzen N (2014). How epigallocatechin gallate can inhibit α-synuclein oligomer toxicity *in vitro*. J. Biol. Chem..

[24] Konijnenberg A (2016). Opposite Structural Effects of Epigallocatechin-3-gallate and Dopamine Binding to a-synuclein. Anal Chem.

[25] Zhao J (2017). (−)-Epigallocatechin-3-gallate (EGCG) inhibits fibrillation, disaggregates amyloid fibrils of α-synuclein, and protects PC12 cells against α-synuclein-induced toxicity. RSC Adv..

[26] Wantyghem J, Baron MH, Picquart M, Lavialle F (1990). Conformational changes of Robinia pseudoacacia lectin related to modifications of the environment: FTIR investigation. Biochemistry.

[27] Bhak G, Lee S, Park JW, Cho S, Paik SR (2010). Amyloid hydrogel derived from curly protein fibrils of a-synuclein. Biomaterials.

[28] Paik SR, Lee JH, Kim DH, Chang CS, Kim J (1997). Aluminum-induced structural alterations of the precursor of the non-A beta component of Alzheimer’s disease amyloid. Arch. Biochem. Biophys..

