# Supplementary material for: EGCG-mediated Protection of the Membrane Disruption and Cytotoxicity Caused by the ‘Active Oligomer’ of α-Synuclein
Source: Sci Rep. 2017 Dec 20;7:17945. doi: 10.1038/s41598-017-18349-z (PMC5738379; doi:10.1038/s41598-017-18349-z)
Supplement: Supplementary file 1 — Supplementary information [file 41598_2017_18349_MOESM1_ESM.pdf]

Supplementary Information

EGCG-mediated Protection of the Membrane Disruption and Cytotoxicity Caused by the 'Active Oligomer' of α-Synuclein

Jee Eun Yang [a], Kun Yil Rhoo [b], Soonkoo Lee [a], Jong Tak Lee [a], Jae Hyung Park [a], Ghibom Bhak [a], and Seung R. Paik [a,b,*]

[a] School of Chemical and Biological Engineering, Institute of Chemical Processes, College of Engineering, Seoul National University, Seoul 08826, Korea (Republic of)

[b] Interdisciplinary program of Bioengineering, College of Engineering, Seoul National University, Seoul 08826, Korea (Republic of)

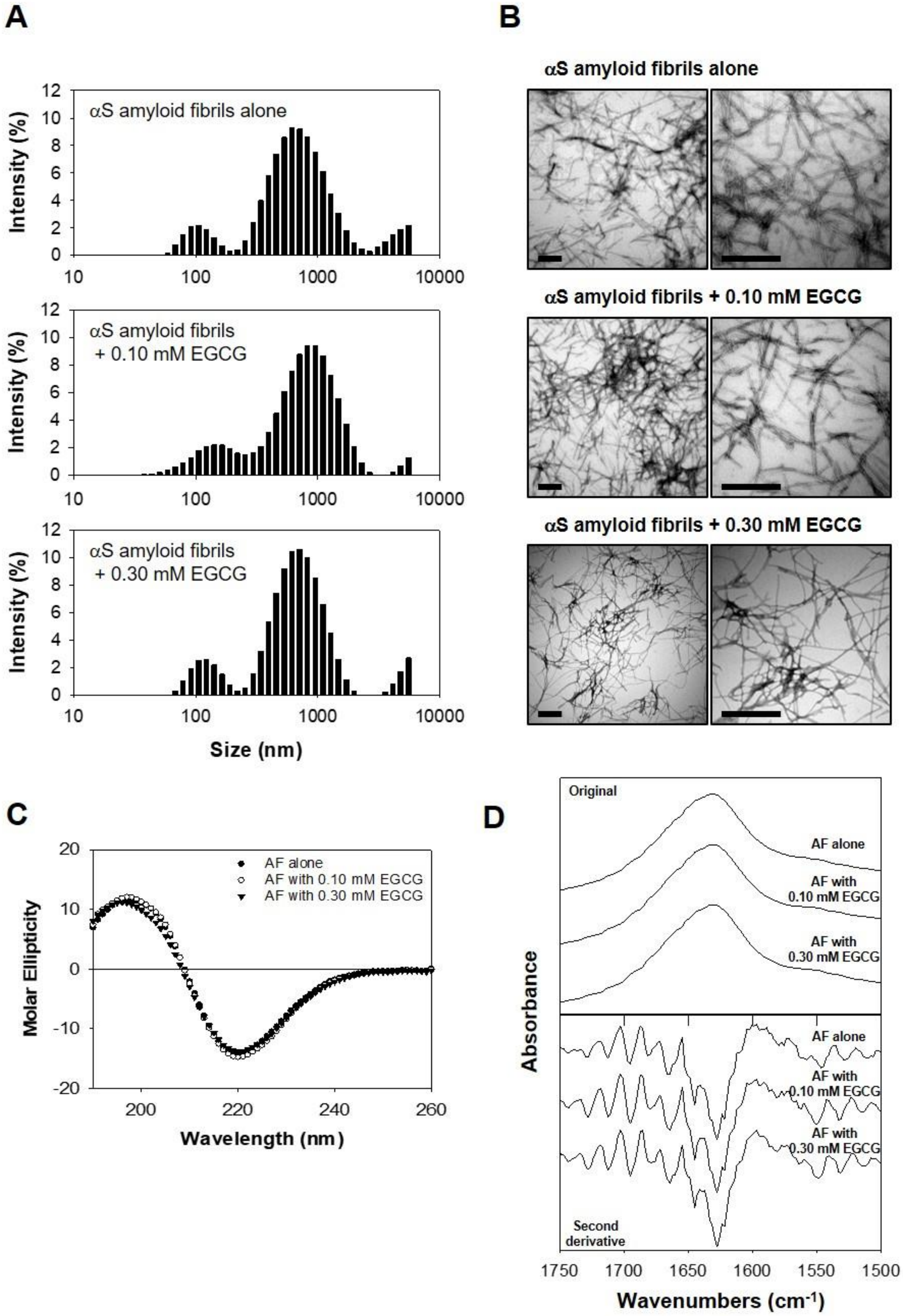
A
αS amyloid fibrils alone
αS amyloid fibrils + 0.10 mM EGCG
αS amyloid fibrils + 0.30 mM EGCG
Intensity (%)
Size (nm)
B
αS amyloid fibrils alone
αS amyloid fibrils + 0.10 mM EGCG
αS amyloid fibrils + 0.30 mM EGCG
C
AF alone
AF with 0.10 mM EGCG
AF with 0.30 mM EGCG
Molar Ellipticity
Wavelength (nm)
D
Original
AF alone
AF with 0.10 mM EGCG
AF with 0.30 mM EGCG
Second derivative
Absorbance
Wavenumbers (cm-1)

Figure S1. Structural integrity of αS amyloid fibrils upon EGCG treatment. After an incubation with EGCG for 1 hr at 37˚C, amyloid fibrils of αS were analyzed with (A) DLS, (B) TEM, (C) CD spectroscopy, and (D) FT-IR spectroscopy. For DLS analysis, the amyloid fibrils of αS were incubated with 0, 0.1, and 0.3 mM EGCG at 37°C for 1 hr at 200 rpm. The resulting mixtures of amyloid fibrils and EGCG were collected as the pellets via a centrifugation at 16,100 × g for 30 min and resuspended with 1 mL of distilled water for the analysis. For Fourier transform infrared (FTIR) spectroscopy analysis, the amyloid fibrils of αS incubated with and without EGCG at 37°C for 1 hr at 200 rpm were collected as the precipitates and placed on ZnSe ATP crystal, and their ATR spectra were monitored with Nicolet 6700 FTIR spectrometer equipped with DTGS detector (Thermo Fisher Scientific Inc.). FTIR absorption spectra after solvent subtraction and their second derivative spectra are shown between 1500-1750 cm^{-1} region. Data fitting and curve deconvolution were performed by OriginPro 2015 software (OriginLab, Northampton).

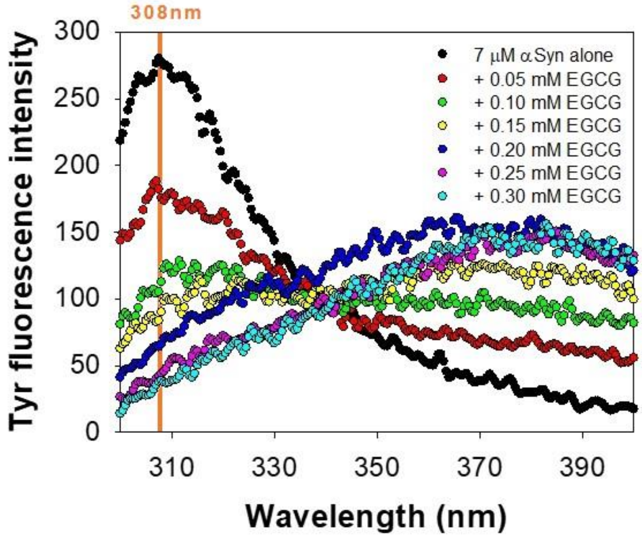

Figure S2. Change in the intrinsic fluorescence of tyrosine residues of αS upon EGCG interaction. The intrinsic tyrosine fluorescence of αS (70 μM) incubated with and without various concentrations of EGCG for 30 min at 37°C was monitored between 300 nm and 400 nm with an excitation at 274 nm. The differences in the fluorescence intensity at 308 nm were used to plot the hyperbolic binding curve (Figure 2A) with EGCG.

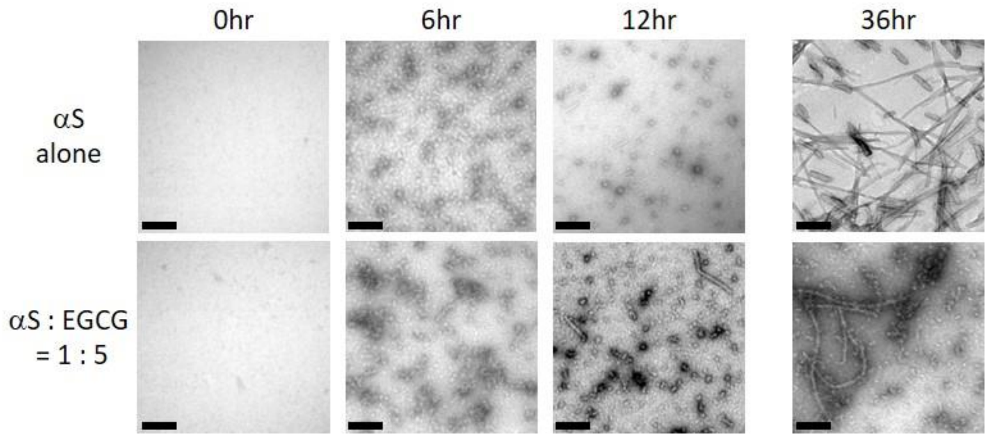

Figure S3. The αS aggregation processes monitored with the images of TEM. The protein aggregates of αS (70 μM) incubated without (upper) and with (lower) 350 μM EGCG at 37°C under a shaking condition are revealed with TEM. The scale bars represent 200 nm.

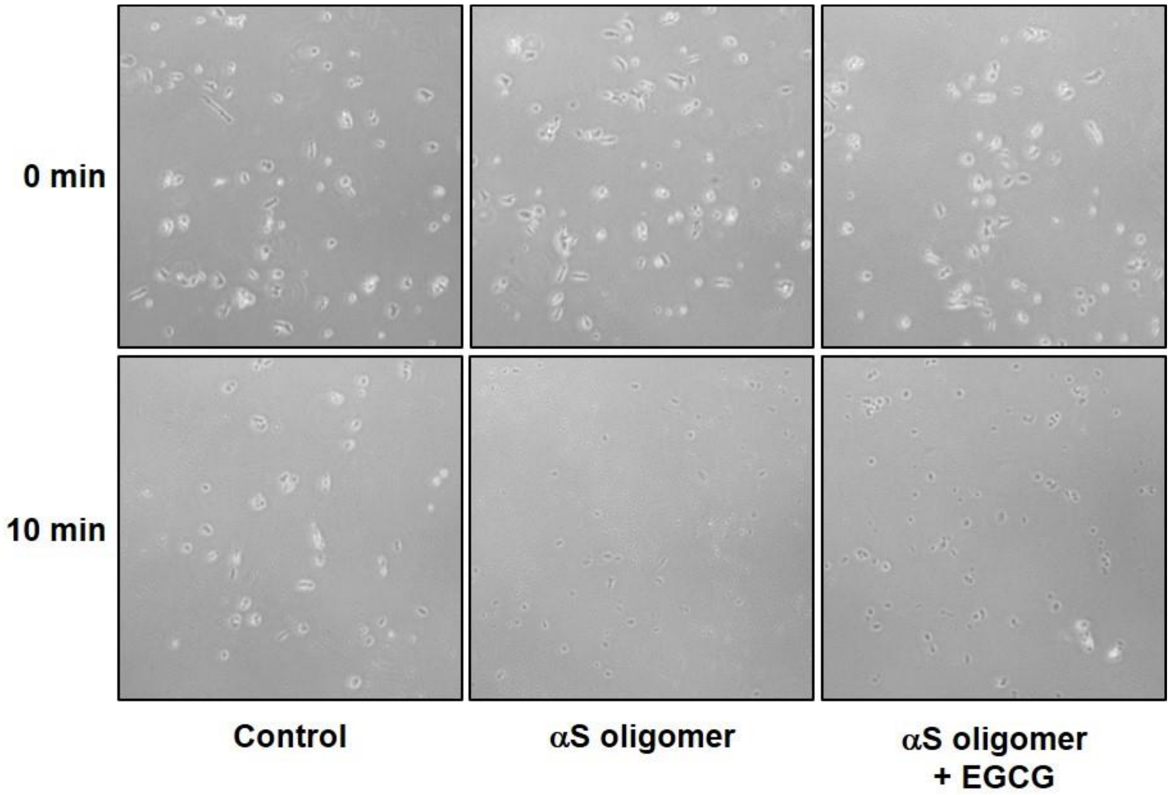

Figure S4. Detachment of SH-SY5Y cells from a glass surface before and after AO treatment. The SH-SY5Y cells remain attached on a glass surface after 10 min incubation at room temperature in the presence and absence of AOs (70 μM) pre-treated with or without EGCG (350 μM).

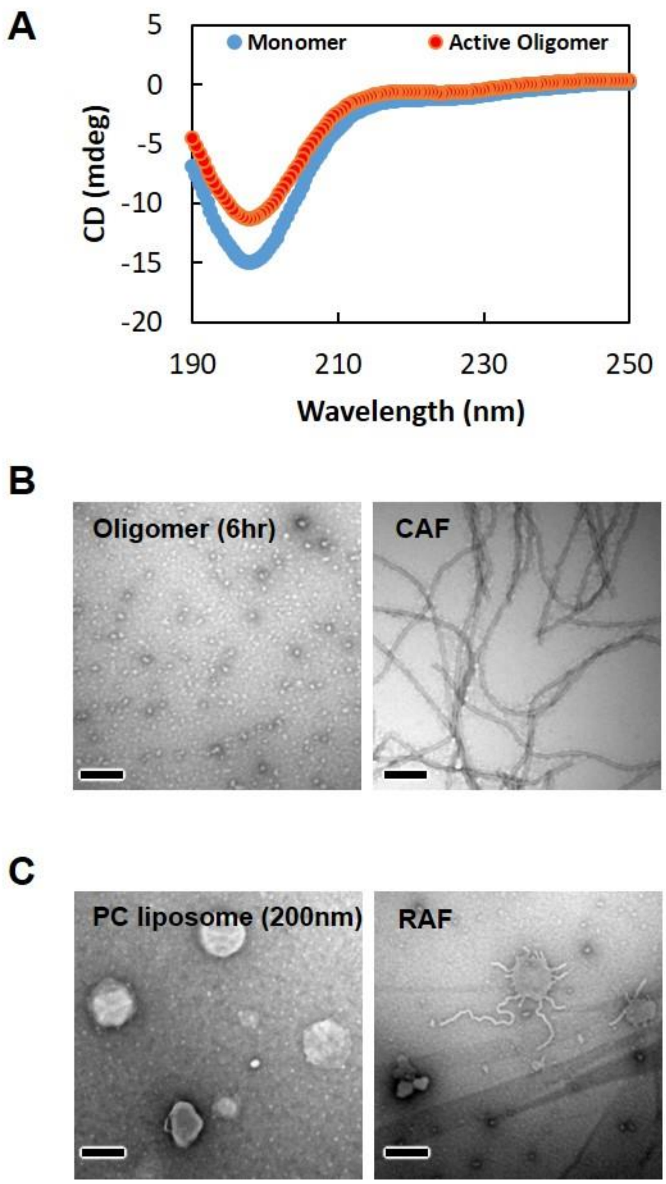

Figure S5. CD spectrum of AOs and their activity producing CAF and RAF. (A) The CD spectrum of AOs in comparison with that of αS monomers. (B) TEM images of AOs (left) and their conversion into CAFs (right) after the repetitive membrane filtration. (C) TEM images of RAFs (right) produced on the surface of PC liposomes (left) after 15 min co-incubation. Scale bars represent 200 nm.

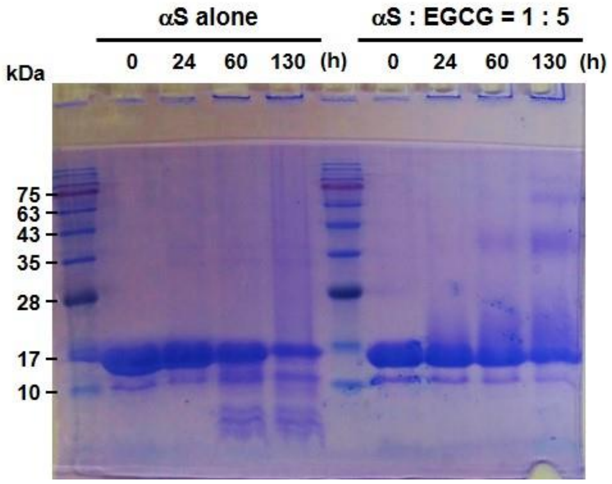

Figure S6. A full-length SDS-PAGE gel for analyzing the αS fibrillation in the absence or presence of EGCG. The gels shown in Figure 1A are cropped from this gel.
